# Oncological outcomes following radical prostatectomy for patients with pT4 prostate cancer

**DOI:** 10.1590/S1677-5538.IBJU.2016.0290

**Published:** 2016

**Authors:** Dharam Kaushik, Stephen A. Boorjian, R. Houston Thompson, Manuel S. Eisenberg, Rachel E. Carlson, Eric J. Bergstralh, Igor Frank, Matthew T. Gettman, Matthew K. Tollefson, R. Jeffrey Karnes

**Affiliations:** 1 Department of Urology, Mayo Clinic, Rochester, Minnesota, USA; 2 Department of Urology, University of Texas Health Science Center, San Antonio, Texas, USA; 3 Division of Biomedical Statistics and Informatics, Mayo Clinic, Rochester, Minnesota, USA

**Keywords:** Prostatic Neoplasms, Prostatectomy, Neoplasm Metastasis

## Abstract

**Objectives:**

Radical prostatectomy (RP) for locally advanced prostate cancer may reduce the risk of metastasis and cancer-specific death. Herein, we evaluated the outcomes for patients with pT4 disease treated with RP.

**Materials and methods:**

Among 19,800 men treated with RP at Mayo Clinic from 1987 to 2010, 87 were found to have pT4 tumors. Biochemical recurrence (BCR)-free survival, systemic progression (SP) free survival and overall survival (OS) were estimated using the Kaplan-Meier method and compared with the log-rank test. Cox proportional hazards regression models were used to assess the association of clinic-pathological features with outcome.

**Results:**

Median follow-up was 9.8 years (IQR 3.6, 13.4). Of the 87 patients, 50 (57.5%) were diagnosed with BCR, 30 (34.5%) developed SP, and 38 (43.7%) died, with 11 (12.6%) dying of prostate cancer. Adjuvant androgen deprivation therapy was administered to 77 men, while 32 received adjuvant external beam radiation therapy. Ten-year BCR-free survival, SP-free survival, and OS was 37%, 64%, and 70% respectively. On multivariate analysis, the presence of positive lymph nodes was marginally significantly associated with patients' risk of BCR (HR: 1.94; p=0.05), while both positive lymph nodes (HR 2.96; p=0.02) and high pathologic Gleason score (HR 1.95; p=0.03) were associated with SP.

**Conclusions:**

Patients with pT4 disease may experience long-term survival following RP, and as such, when technically feasible, surgical resection should be considered in the multimodal treatment approach to these men.

## INTRODUCTION

In 2016 there will be approximately 180,890 new cases of prostate cancer and 26.120 related deaths in the US (1). Despite the noted stage migration in prostate cancer over the course of the PSA era, approximately 10% of patients present with locally advanced disease, which increases the risk of disease progression and mortality after initial treatment (2). Historically, the majority of patients with high-risk prostate cancer have been managed with external beam radiation therapy (EBRT) and androgen deprivation therapy (ADT) (3–6).

More recently, several observational series have demonstrated the technical feasibility of radical prostatectomy (RP) in the setting of high-risk disease and have reported 5-year progression-free survival rates of approximately 85% (7–10). While in high-risk disease, surgery is often a component of a multimodal treatment approach; RP provides accurate pathologic staging of both the primary tumor and pelvic lymph nodes and may afford durable local control. Of note, the majority of surgical series on locally advanced disease have consisted of patients with either clinical or pathologic T3 tumors. Indeed, the treatment outcomes and prognostic variables for patients with pT4 prostate cancer remain poorly described. Here, we evaluated our experience with RP for patients in whom the final pathologic analysis demonstrated pT4 disease. Specifically, we analyzed the long-term rates of biochemical recurrence (BCR), systemic progression (SP) and overall survival (OS) following surgery as part of a multimodal treatment approach. Furthermore, we identified clinico-pathologic variables associated with oncological outcome in these men.

## MATERIALS AND METHODS

### 

#### Patient selection

After Institutional Review Board approval (#12-007416) was obtained, we reviewed our Prostatectomy Registry and identified 19,800 patients who underwent RP between 1987 and 2010. Surgical procedures were performed using standard techniques. For the uniformity of diagnosis and staging, all the cases were reviewed by central pathology laboratory and surgical specimens were processed according to standard pathological procedures and staged according to the 2009 American Joint Committee on Cancer staging system for prostate cancer (11). A total of 87 men were found to have pT4 disease, and form the study cohort here. Out of these, 7 patients underwent robotic radical prostatectomy with pelvic lymph node dissection (PLND) and 80 underwent radical retropubic prostatectomy with PLND. Pelvic lymph node dissection was performed utilizing the following template-we completely removed all lymph node tissue along the external iliac vein, the distal limit being the deep circumflex vein and the femoral canal. We removed all fibrofatty tissue from the obturator fossa to completely skeletonize the obturator nerve. Proximally, PLND was performed up to and including the bifurcation of the common iliac artery. The lateral limit consisted of the pelvic sidewall, and the medial dissection limit was defined by perivesical fat. In high-risk patients included in our cohort, LNs along the internal iliac vessels were dissected.

The retrospective nature of our dataset precludes a standardized approach to surveillance; however, postoperative assessments, including physical examination and serum PSA measurement, were generally done quarterly for the initial 2 years after surgery, semi-annually for an additional 2 years, and annually thereafter. Adjuvant therapy was defined as treatment received ≤90 days of RP, and was given at the discretion of the treating physician, while salvage therapy was defined as treatment received >90 days after RP, and was likewise administered based on clinician's discretion. BCR was defined as a PSA level of ≥0.4ng/mL (12). SP was defined as demonstrable metastasis on radionuclide bone scan or on biopsies outside the prostatic bed. Vital status was identified from death certificates or physician correspondence. For patients followed elsewhere, the Mayo Clinic Prostatectomy Registry prospectively monitors outcomes annually by correspondence.

### Statistical analysis

Continuous features were summarized with medians and interquartile ranges (IQR); categorical features were summarized with frequency counts and percentages. The Kaplan-Meier method was utilized to estimate BCR-free survival, SP-free survival and OS, with differences assessed with log-rank test. Patients were censored at last follow-up or death if the endpoint of interest had not been attained. Cox proportional hazards model was used to estimate the association of clinic-pathologic variables with patient's risk of BCR, SP, and all-cause mortality. Statistical analysis was done using SAS®, version 9.2. All tests were two-sided, with p≤0.05 considered to indicate statistical significance.

## RESULTS

Of 19,800 patients who underwent RP during the time period of study, we identified 87 (0.43%) patients with pT4 disease. Median age at surgery in these men was 65 years (IQR 58, 69). Median preoperative PSA was 12.2ng/mL (IQR5.7, 34.9). Table-1 lists the clinic-pathological features for this cohort. Moreover, 30 (34.5%) and 4 (4.6%) patients received androgen deprivation therapy (ADT) and external beam radiation therapy (EBRT), respectively, prior to RP.

**Table 1:** Clinical and pathological features of pT4 prostate cancer patients.

		Total (n=87)
**Clinical T Stage**		(n=85)
	T1c	15(17.6%)
	T2a	20(23.5%)
	T2b	11(12.9%)
	T3/4	39(45.9%)
**Biopsy Gleason Score**		(n=54)
	≤6	13(24.1%)
	7	14(25.9%)
	8-10	27(50.0%)
**Pathological Gleason Score**		(n=79)
	≤6	12 (15.2%)
	7	32 (40.5%)
	8-10	35 (44.3%)
**Seminal vesicle invasion**		(n=86)
	No	24 (27.9%)
	Yes	62 (72.1%)
**Nodal status**		(n=87)
	Negative	47 (54.0%)
	Positive	40 (46.0%)
**Positive surgical margin**		(n=87)
	No	5 (5.7%)
**Age at Surgery**		
	N	(n=87)
	Median	65.0 (IQR: 58-69)
**Pre-op PSA (ng/mL)**		
	N	(n=76)
	Median	12.2 (IQR: 5.7-34.9)
**Race**		(n=87)
	Caucasian	67 (77.0%)
	Other	1 (1.1%)
	Undetermined	19 (21.8%)

**Figure 1:**
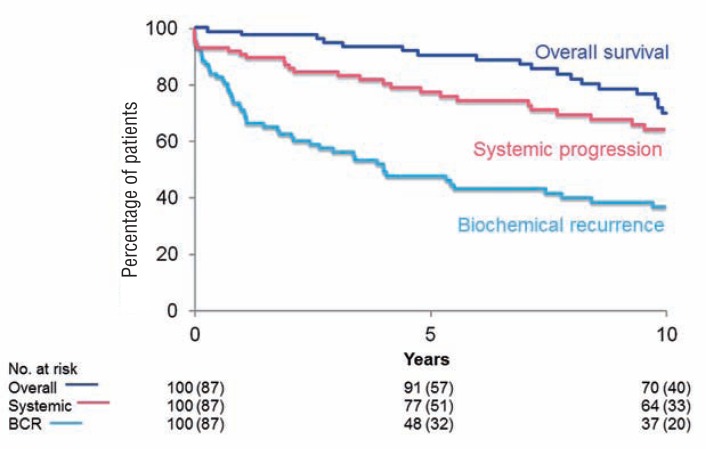
Kaplan-Meier plot showing 10-year Overall Survival, Systemic progression and biochemical recurrence.

Median follow-up after RP was 9.8 years (IQR 3.6, 13.4), during which time 50 patients experienced BCR, 10 were diagnosed with a local recurrence, 30 developed SP and 38 died, with 11 dying of prostate cancer. A total of 77 men were treated with adjuvant ADT, while 32 received adjuvant EBRT. We noted that the 10-year BCR-free survival in these patients was 37%, while 64% were free from SP and the overall survival was 70% (Figure-1).

**Figure 2:**
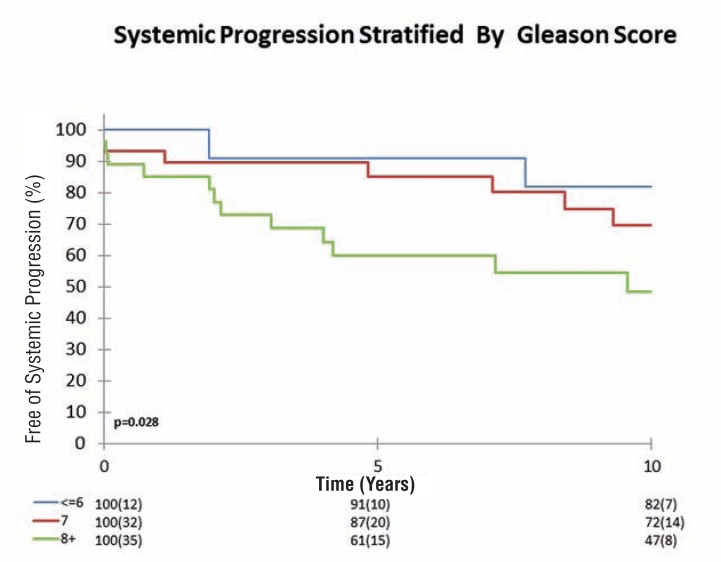
Kaplan-Meier plot showing Systemic-progression stratified by Gleason-score.

**Figure 3:**
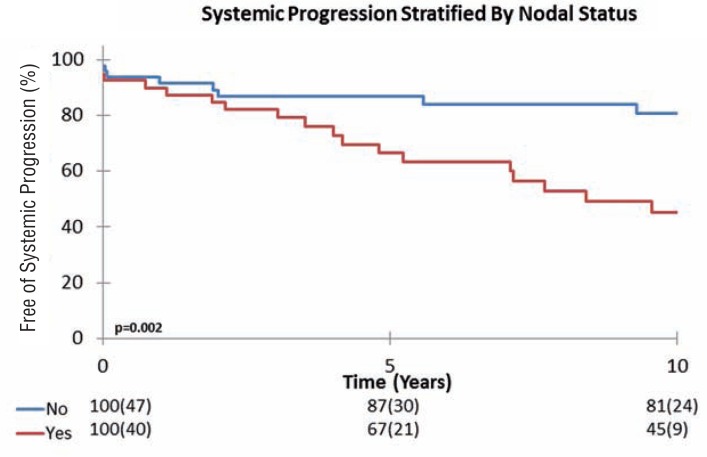
Kaplan-Meier plot showing Systemic-progression stratified by nodal status.

We then further stratified patient's risk of SP by pathological Gleason score, and lymph node status. As such, the Kaplan-Meier analysis with the log rank test showed that for patients with pT4 tumors, both higher Gleason score and positive lymph node status showed differences in progression between the groups. The 10-year SP-free survival for patients with a pT4 Gleason 8-10 tumor was 47%, versus 72% for patients with a Gleason 7 tumor and 82% for patients with Gleason 6 disease (p=0.028) (Figure-2). Likewise, the 10-year SP-free survival for patients with positive lymph nodes was 45%, versus 81% for pT4N0 tumors (p=0.002) (Figure-3).

Moreover, on multivariate analysis (Table-2), positive lymph node status was found to be marginally significantly associated with patient's risk of BCR [HR 1.94, p=0.05], while both positive lymph nodes [HR 2.96, p=0.02] and higher pathological Gleason score [HR 1.95, p=0.03] were associated with a significantly increased risk for systemic progression in these men. Ten-year survival estimates for BCR, SP and OS grouped by pathological Gleason Score, median prior PSA and receipt of prior treatment are shown in Table-3.

**Table 2:** Multivariate analysis of factors associated with biochemical recurrence, systemic progression and all-cause mortality.

	Biochemical Failure (46 events)			Systemic Progression (27 events)				Death (29 events)	
	**HR**	**95% CI**	**p-value**	**HR**	**95% CI**	**p-value**	**HR**	**95% CI**	**p-value**
Path Gleason Score Group	1.17	(0.78-1.75)	0.42	1.95	(1.04-3.60)	0.03	1.06	(0.61-1.81)	0.83
Log 2 preoperative PSA (doubling)	1.13	(0.95-1.36)	0.15	0.94	(0.74-1.19)	0.65	1.14	(0.88-1.47)	0.30
Treatment prior to RP	1.62	(0.89-2.94)	0.11	1.85	(0.83-4.13)	0.12	1.003	(0.44-2.28)	0.99
Positive lymph nodes	1.94	(1.01-3.72)	0.05	2.96	(1.20-7.29)	0.02	1.37	(0.60-3.11)	0.44
Seminal vesicle invasion	1.36	(0.64-2.87)	0.42	3.91	(0.86-17.63)	0.07	1.76	(0.62-5.03)	0.28

**Table 3:** Survival estimates for biochemical recurrence, systemic progression and overall survival.

		10-year Survival Estimates for Biochemical recurrence (%)	10-year Survival Estimates for Systemic Progression (%)	10-year Survival Estimates for Overall Survival (%)
**Path Gleason Score**				
	<=6	47	82	73
	7	42	72	78
	8+	24	47	68
**PSA (ng/DL)**				
	<=12.2	41	64	76
	>12.2	35	66	68
**Prior Treatment**				
	No	49	73	70
	Yes	11	45	69

## DISCUSSION

We report the natural oncological outcomes of patients with pT4 disease treated with RP. In this cohort, we found that over one-third of patients remained free of BCR at 10 years after surgery, while the 10-year overall survival for these men was 70%. In addition, we noted that both high pathologic Gleason score and lymph node involvement contributed to the development of metastatic disease in these men.

While the outcomes for patients with locally-advanced prostate tumors treated with surgery have been previously described, (7, 10, 13) these series have primarily focused on T3 tumors and, as such, the present study represents what is, to our knowledge, the first report to specifically focus on pT4 prostate cancer treated with RP. Likewise, while prior series have reported long-term survival following surgery in patients with lymph-node positive disease (14), our data further provide evidence of a role for surgery in the setting of very high-risk prostate cancer. Indeed, even in patients with pT4N1 disease, the highest-risk patients, nearly half of such men were without evidence of clinical metastases at 10 years after RP.

Level-1 data exist regarding the role of EBRT and androgen deprivation therapy (ADT) in management of locally advanced prostate cancer. In Bolla et al. trial, out of 415 patients, 89% were high risk (cT3/T4) with reported 5 year-OS in combined EBRT/ADT group to be 79% compared to 62% in the radiotherapy group alone (5). In the SPCG-7/SFUO-3 trial, 880 patients, predominantly cT3N0M0, were randomly assigned to either ADT or ADT with EBRT. Ten-year OS was better in the EBRT/ADT arm compared to ADT only arm (70% vs. 60%) (3). Both these trials provided evidence supporting addition of local radiotherapy to endocrine treatment had an important effect on overall and cancer-specific mortality in locally advanced prostate cancer. Importantly, these studies confirm the critical role of local tumor control in addition to systemic therapies.

Because of the heterogeneous population of locally-advanced prostate cancer and lack of data from randomized trials comparing RP and EBRT, definitive inferences cannot be reached regarding the relative effectiveness of each treatment modality for achieving long-term cancer control. There have been few retrospective comparative studies; while 2 studies have shown superior biochemical relapse-free survival with EBRT in comparison to RP, (15, 16) one study has shown better metastasis-free survival (17) and another studying showing better overall survival with RP compared to EBRT/ADT (18).

Interestingly, Thompson et al. evaluated 1,286 men with metastatic disease from the Southwest Oncology Group Study 8894 and determined that patients who underwent RP prior to developing metastatic disease had lower risk of death than patients who did not (HR 0.77 [0.53, 0.89]) (19). Multiple hypotheses may be offered to support a benefit for RP in advanced stage prostate cancer, including prevention of development of metastatic disease from primary tumor (19).

Zelefsky et al. (17) reviewed a cohort of 2,380 patients who either underwent RP or EBRT and evaluated them for systemic progression-free survival and cancer-specific survival (CSS). On their multivariate analysis, RP was found to be associated with reduced risk of developing metastatic disease in comparison to EBRT, especially in high-risk patients (HR 0.35; p<0.001).

In addition, as the management for patients with locally advanced prostate cancer is likely to involve a multi-modal approach, RP as the initial treatment affords accurate pathologic staging, which may thereby guide the selective application of secondary therapy. That is, up to 25% of patients with clinical T3 tumors in fact have organ-confined disease at surgery (20). As such, RP may facilitate the identification of patients with pathologic extraprostatic disease who might benefit from adjuvant RT (21) as well as patients with positive lymph nodes, who might benefit from adjuvant ADT (22). Further, as the extension of locally advanced tumors may result in debilitating loco-regional symptoms including recurrent hematuria, pelvic pain, as well as urinary, rectal and ureteral obstruction, the durable local control with surgery noted here (only 11.5% of the patients have experienced a local recurrence) may improve patient's quality of life as well. Indeed, a higher rate of hospital admissions, rectal or anal procedures, and open surgical procedures has been noted in patients treated with radiotherapy for prostate cancer versus patients treated with surgery (23).

Primary RP with PLND remains the only method that provides conclusive pathological evidence and excellent loco-regional control as shown by low local recurrence rate of 11.5% in our study. With data from the Southwest Oncology Group (SWOG 8794) for the use of adjuvant radiation therapy in high-risk patients (21), and Messing trial results demonstrating an advantage in survival for long-term ADT in lymph node-positive patients (22), good pathologic data are an important step toward multi-modality approach. Admittedly, the optimal management for patients with locally advanced prostate cancer remains to be determined, ideally in a prospective clinical trial setting. Nevertheless, there remains an absence of comparative level I evidence. We recognize that our study is limited by its retrospective design. Further, although the entity of pT4 disease at RP is uncommon, we acknowledge the relatively small patient sample size here. Likewise, it must be acknowledged that this cohort represents a highly selective cohort of patients, and, as noted above, the optimal treatment for such men remains to be determined. Nevertheless, we believe that surgery represents a component of the often multi-modal approach for locally advanced prostate cancer, and may be associated with favorable long-term survival.

In conclusion, radical prostatectomy with pelvic lymph node dissection in the setting of locally advanced prostate cancer is associated with durable loco-regional control and definitive pathologic staging, which in turn facilitates the selective application of secondary therapies. As such, surgical resection should continue to be considered in the multi-modal treatment approach to these men.
